# Job satisfaction and its associated factors among optometrists in Ghana: a cross-sectional study

**DOI:** 10.1186/s12955-020-01650-3

**Published:** 2021-01-07

**Authors:** Kwadwo Owusu Akuffo, Eldad Agyei-Manu, David Ben Kumah, Anthony Danso-Appiah, Abubakar Sadik Mohammed, Akosua Kesewah Asare, Emmanuel Kofi Addo

**Affiliations:** 1 Department of Optometry and Visual Science, College of Science, Kwame Nkrumah University of Science and Technology, Kumasi, Ghana; 2 Usher Institute for Population Health Sciences and Informatics, College of Medicine and Veterinary Medicine, University of Edinburgh, Edinburgh, UK; 3 Department of Epidemiology and Disease Control, School of Public Health, University of Ghana, Legon, Ghana; 4 University of Ghana Centre for Evidence Synthesis and Policy, School of Public Health, University of Ghana, Legon, Ghana; 5 Department of Ophthalmology and Visual Sciences, University of British Columbia, Vancouver, Canada; 6 Department of Ophthalmology and Visual Sciences, Moran Eye Centre, University of Utah, Salt Lake City, Utah USA; 7 Department of Nutrition and Integrative Physiology, University of Utah, Salt Lake City, Utah USA

**Keywords:** Determinants, Factors, Healthcare professionals, Ghana, Job, Job satisfaction, Optometrist, Satisfied

## Abstract

**Background:**

Job satisfaction describes an employee’s motivation and/or feeling of satisfaction towards his/her work. Globally, healthcare professionals’ turnover and retention play a critical role in the delivery of essential health services. In Ghana, however, little has been done to ascertain job satisfaction levels among human resources for eye-health. The objective of this study therefore was to assess job satisfaction and its associated factors among optometrists in Ghana.

**Methods:**

A cross-sectional survey was conducted among 304 registered and licensed optometrists of the Ghana Optometric Association between September 2018 and June 2019. A validated, well-structured questionnaire was used to elicit information on socio-demographic characteristics of participants and measures on job satisfaction. Scores from a five-point Likert scale was employed to examine job satisfaction and its associated factors. Linear regression analyses were used to evaluate the association between overall job satisfaction and its associated factors using Rasch logit scores.

**Results:**

A total of 214 optometrists gave valid responses to the questionnaires used for the final analysis. The mean (± SD) score of the overall perception of job satisfaction among optometrists was 3.36 (± 1.00), with 74.3% of them being satisfied with their jobs. After statistical adjustment, Good work-life balance (Unstandardized co-efficient (β) = 0.288, *p* = 0.001), Salary (β = 0.222, *p* < 0.0005), Supervision (β = 0.117, *p* = 0.044), and Continuing Education Opportunities (β = 0.138, *p* = 0.017) were all significantly associated with higher levels of overall job satisfaction.

**Conclusions:**

Most optometrists were satisfied with their jobs. Effective strategic planning and management of human resources for eye-health in Ghana are essential in the development of quality eye-health systems and the provision of high-quality eyecare services.

## Background

Over the years, healthcare professionals’ retention and satisfaction have played a critical role in the delivery of essential healthcare services in any organization worldwide. Job satisfaction, a multidimensional concept, highlights the level of an employee’s contentment and motivational drive towards achieving organizational goals [1, 2]. A systematic review by Willis-Shattuck, Bidwell [3] showed that healthcare professionals’ retention and turnover in developing countries such as Ghana, has become a major threat to achieving access to quality health care, as enshrined in the Sustainable Development Goal 3 (good health and well-being). The diverse factors which are considered a sine qua non for job satisfaction among different healthcare professionals [4], if adequately satisfied, provide the right environment for utmost productivity. In Ghana, a survey by Bonenberger et al. [5] on turnover intentions by healthcare professionals such as doctors, nurses, pharmacists and allied healthcare professionals (e.g. optometrists) showed that 69% of them had intentions of exiting their health facilities due to low levels of job satisfaction.

The theories that explain job satisfaction provide the basis to identify the various factors which influence job satisfaction and suggest ways of improving employee’s job satisfaction. These theories include the Herzberg’s motivator-hygiene theory [6], the Maslow’s needs hierarchy theory [7], the dispositional approach [8], and the Job Characteristics Model [9]. Although little empirical evidence has been documented for the Maslow’s needs hierarchy and the Herzberg’s motivator-hygiene theories [10], the Job Characteristics Model and dispositional approach continue to accumulate empirical evidence to suggest that job satisfaction among employees is usually affected by psychological and personality factors [11]. Nevertheless, motivational factors tend to influence the immediate job environment and subsequently impact how employees are satisfied with their jobs.

Diverse studies have shown that there are various determinants for job satisfaction among healthcare professionals such as optometrists, physicians, nurses, etc. [12–14]. These factors, which include salary, job security, supervision, supportive working environment, etc. [3, 4, 15], ultimately considerably impact the quality of health delivery services and may lead to low productivity [16]. In Ghana, it has been shown that poor working conditions such as low financial incentives, poor working environment, unavailability of resources, and lack of opportunities for career development lead to job dissatisfaction among healthcare professionals, including optometrists, and consequently lead to increased turnover and low patient care [17].

There have been concerns about optometrists’ job satisfaction due to their crucial role in the delivery of quality eyecare services to patients [18–20] and the potential for optometrists’ turnover [14]. As primary eyecare providers, optometrists provide essential vision-related services such as refractive correction [21, 22], detection and management of eye diseases [23], as well as specialized eyecare services such as low vision rehabilitation [24], vision therapy [25], and contact lens fitting [26]. These essential eye health services have contributed significantly to achieving VISION 2020 (eliminating avoidable visual impairment and blindness), especially in sub-Saharan Africa [27, 28]. In Ghana, however, optometrists play a critical role in the delivery of comprehensive refractive error services. As opposed to other eyecare cadres such as ophthalmologists and ophthalmic nurses, optometrists primarily undergo comprehensive training in visual function and optical technology services, which is aimed at correcting people with refractive defects in their vision/visual system [29]. Refractive errors have been shown to increase the burden of avoidable blindness and considerably impact the quality of life of affected individuals. Notably, a systematic review and meta-analysis by Hashemi et al. [30] provided evidence that the prevalence of myopia, hyperopia, and astigmatism among adults in Africa was 11.4%, 38.6%, and 16.2%, respectively, as opposed to 14.2%, 3.0%, and 6.2%, respectively in children. The central role of optometrists in the provision of quality refractive error services in Ghana is inevitably needed in reducing the burden of avoidable visual impairment and blindness in the country [31].

In spite of this significant role by optometrists in Ghana’s eyecare industry, little or no study has been conducted to assess their job satisfaction level and provide evidence base for policy direction in addressing the growing needs of optometrists in the country. The World Health Organization’s (WHO) “Global Strategy on Human Resources for Health: Workforce 2030” sets out the policy agenda to ensure a workforce that is fit for purpose to attain the targets of the Sustainable Development Goals (SDGs). This agenda considers the emerging evidence that addressing the job challenges faced by health workers and investments in the health workforce, including optometrists, could further generate socio-economic development and sustainable economic growth. Low morale among the optometric workforce in Ghana may undermine the quality of specialized eyecare services provided to the populace. This study therefore sought to assess the level of job satisfaction among optometrists in Ghana and its associated determinants. This important novel study would provide primary data (for the first time) to assist the development of human resource policies regarding optometric practice and healthcare administration in Ghana.

## Methods

### Study aim, design, setting, and population

This cross-sectional survey, conducted between September 2018 and June 2019, assessed the factors affecting job satisfaction among optometrists in Ghana. Ghana is a lower-middle income country (a country with a total economic value between $1006 and $3955) [32] in the sub-Saharan African region (West Africa), and shares borders with Cote D’Ivoire (in the west), Burkina Faso (in the north), Togo (in the east), and the Gulf of Guinea (in the south). The country has a population of about 30 million people with about 2.15% growth rate, and a total land area of about 238,533 km^2^ [32]. As at the commencement of this study, Ghana was broadly divided into 10 regions (currently 16 regions), namely the Greater Accra, Central, Western, Eastern, Brong-Ahafo, Ashanti, Volta, Northern, Upper East, and Upper West regions. The study population encompassed all registered optometrists of the Ghana Optometric Association (GOA), across all regions of the country. GOA is the professional body responsible for the advancement of optometry in Ghana and harmonizes the activities of optometrists with the Allied Health Professions Council of Ghana (AHPC).

### Study participants

An official letter was sent to the GOA requesting permission to conduct the study. The letter also stated the purpose of the study. Upon review and further correspondence with the GOA, formal communication was sent by the leadership of the GOA to all its members across the country, informing them about the study and encouraging members to participate. GOA subsequently provided a professional registry of all its members. The professional registry of GOA in 2018, as at the commencement of the study, had 406 registered optometrists. However, on further review of the GOA’s database, the study investigators excluded optometrists who had no/wrong contact information/details (email addresses and/or telephone numbers) or were currently working outside Ghana. Subsequently, a total of 304 optometrists were eligible for the study and were contacted via email or visit to participate in the study. However, a total of 214 optometrists responded to the study by completing study questionnaires; representing a participation rate of 70.4%.

### Data collection

A validated, well-structured questionnaire adapted from Paudel et al. [33] with both open- and closed-ended questions was administered to all the participants. The questionnaires were administered either through a face-to-face interview or via email (google form) [34], after explaining instructions and essential terms to the participants. The details of both softcopy and hardcopy questionnaires were the same. The questionnaire was composed of two sections. The first section (Part A), comprising 21 items, elicited information on the socio-demographic characteristics of participants such as age, sex, marital status, number of children (if any), highest educational level, location of workplace, practice setting, working hours per week, work experience, good-work-life balance, first job appointment, and duration for a first job appointment. The second section (Part B), comprising 15 items, elicited information on the 14 factors responsible for participants’ level of satisfaction with their current job, as well as their overall perception on the level of job satisfaction (item 15 in the second section). The factors assessed in this section included salary; non-financial incentives (e.g. vacation, sick leave, etc.); job security; workplace equipment and facilities; supervision; encouragement, reward and positive feedback from institution; recognition by co-workers, level of job responsibility; task variety; workload; level of control over job; support from co-workers; continuing education opportunities; and opportunities for career advancement. A five-point Likert scale was used to assess all 15 items in the second section; 1—very dissatisfied, 2—dissatisfied, 3—neither satisfied nor dissatisfied, 4—satisfied, and 5—very satisfied. Each face-to-face interview generally lasted for about 20 min whereas the filling of online questionnaires lasted for about 10 min.

### Ethical approval

The study adhered to the Declaration of Helsinki and was approved by the Committee on Human Research Publication and Ethics of the Kwame Nkrumah University of Science and Technology (KNUST), College of Health Sciences (CHRPE/AP/034/19). Permission was obtained from the GOA. Written informed consent was obtained from all participants after explaining the objectives, nature, method and importance of the study to them.

### Statistical analysis

The data obtained were analysed using Statistical Product and Service Solution (IBM Corporation IBM® SPSS® Statistics for Windows, Version 25.0 Armonk, NY) compatible with Windows 10. Frequencies and percentages of demographic variables, as well as the determinants of job satisfaction were analyzed using descriptive statistics.

For this study’s analyses, the overall perception of each participant’s job satisfaction was calculated by finding an average score of the 14 items (factors) for job satisfaction, and subsequently classified job satisfaction among participants into two groups based on the average scores obtained: *Satisfied* (having an average score greater than 3) and *Not satisfied* (having an average score less than or equal to 3). Also, the average of the scores for self-reported overall job satisfaction by all participants (item 15 in the second section of questionnaire) was calculated.

Rasch logit scores were employed in bivariate simple linear and multivariate linear regression models. Only variables found to be significantly associated with overall level of job satisfaction (*p* < 0.05) in bivariate models were selected for inclusion in multivariate linear regression analyses.

## Results

A total of 121 (56.5%) completed online questionnaires and 93 (43.5%) completed printed questionnaires were obtained at the end of the study.

### Socio-demographic characteristics of participants

The mean (± standard deviation [SD]) age of all participants was 33.1 ± 6.0 years (with age range 24–60 years). Majority of the participants were males (69.6%), married (57.0%) and aged 31–45 years (60.7%). Most optometrists worked in the urban setting (81.3%) and the private practice setting (44.9%). A greater proportion of participants (81.8%) had obtained a Doctor of Optometry (OD) degree as their highest level of education, with most of them having about 6–10 years of work experience (50.9%). Table 1 shows the socio-demographic profile of the participants.

**Table 1:** Demographic profile of participants

Characteristic	n (%)
Age (years)	
≤ 30	74 (34.6)
31–45	130 (60.7)
46–60	10 (4.7)
Sex	
Male	149 (69.6)
Female	65 (30.4)
Highest Educational level	
Post-graduate Diploma (optometry)	2 (0.9)
Doctor of Optometry	175 (81.8)
Fellowship	2 (0.9)
Masters	28 (13.1)
Doctor of Philosophy	7 (3.3)
Marital Status	
Single	85 (39.7)
Married	122 (57.0)
Divorced	7 (3.3)
Number of children	
0	106 (49.5)
≥ 1	108 (50.5)
Region of workplace	
Greater Accra	84 (39.3)
Western	10 (4.7)
Central	21 (9.8)
Eastern	14 (6.5)
Ashanti	59 (27.6)
Brong Ahafo	10 (4.7)
Volta	8 (3.7)
Northern	5 (2.3)
Upper East	2 (0.9)
Upper West	1 (0.5)
Location of workplace	
Urban	174 (81.3)
Rural	40 (18.7)
Practice Setting	
Government	62 (29.0)
CHAG/NGO	42 (19.6)
Private	96 (44.9)
Academic	11 (5.1)
Others	3 (1.4)
Working hours per week	
0–40	151 (70.6)
≥ 41	63 (29.4)
Work experience (years)	
0–5	82 (38.3)
6–10	109 (50.9)
≥ 11	23 (10.7)
Good work-life balance	
Yes	169 (79.0)
No	45 (21.0)
First job appointment	
Yes	88 (41.1)
No	126 (58.9)
Duration before first job appointment	
Within 3 months	150 (70.1)
3–6 months	17 (7.9)
6–12 months	25 (11.7)
> 1 year	22 (10.3)
*Routine task areas	
Clinical examination, management, referral	205 (95.8)
Refraction	142 (98.6)
Optical dispensing	211 (66.4)
Contact lens fitting	63 (29.4)
Low vision care	51 (23.8)
Community outreaches	161 (75.2)
Research activities	49 (22.9)
Diagnostic unit	80 (37.4)
*Reason for choosing optometry	
Only study opportunity after school	11 (3.0)
Parental/family influence	8 (2.2)
Very interest in health and eyecare	167 (45.9)
Provide voluntary assistance to the needy and NGOs	48 (13.2)
To earn good income	69 (19.0)
I believe I can become an eye doctor	47 (12.9)
Did not know what else to do after school	8 (2.2)
Other	6 (1.6)
Ownership/Partnership with an established private practice	
Yes	55 (25.7)
No	159 (74.3)
Opportunity to choose another career when given a choice	
Yes	81 (37.9)
No	133 (62.1)

n (%), frequencies and percentages of participants; *n ≠ 214 (multiple responses)

### Distribution/nature of optometric workforce and practice

Most optometrists were working in the Greater Accra (39.3%) and Ashanti (27.6%) regions of Ghana (southern sector of the country), whereas a limited number of them worked in the Upper West (0.5%) and Upper East (0.9%) regions of Ghana (northern sector of the country). A large proportion of optometrists were working in Municipal/District Hospitals (32.2%), while a few optometrists worked in Regional (6.1%) and Tertiary (7.0%) hospitals. Less than half of the total participants (41.1%) were currently working in their first job appointment, and 70.1% of all participants had obtained their first job appointment within three months after optometry study. With reference to clinical practice, the following were recorded for various ophthalmic procedures being undertaken by participants at their workplaces: refraction (98.6%); clinical examination, management and referral (95.8%); optical dispensing (66.4%); contact lens fitting (29.4%); low vision care (23.8%); community outreach (75.2%), research activities (22.9%), and diagnostic unit (37.4%) (see Table 1).

### Motivation for choosing optometry practice

Concerning the reasons why participants chose to offer optometry at the tertiary level, most optometrists (45.9%) reported that they were very interested in health and eyecare. The other reasons given were as follows; 19.0% reported that they wanted to earn good income, 13.2% reported that they wanted to provide voluntary assistance to the needy and NGOs, 12.9% reported that they believed they could become eye doctors, 3.0% reported that optometry was their only opportunity to study after school, 2.2% reported that they were influenced by their parents/family to choose the optometry career, 2.2% reported that they did not know what else to do after secondary school, and 1.6% gave other reasons for choosing optometry at the tertiary level. A few optometrists (25.7%) had their own/partnered established private practice in Ghana. However, majority of them (62.1%) were unwilling to choose another career when given the opportunity to start over again (see Table 1).

### Level of job satisfaction

The mean (± SD) score for the overall perception of job satisfaction reported by optometrists in Ghana was 3.36 ± 1.00, whereas the mean (± SD) of the calculated score (an average of the 14 factors associated with job satisfaction) for the overall perception of job satisfaction among optometrists in Ghana was 3.37 ± 0.73. Thus, the level of job satisfaction reported by participants was comparable to our calculated overall perception of job satisfaction. Most participants (74.3%) reported that they were generally satisfied with their current jobs, whereas 25.7% of respondents were generally not satisfied with their current jobs. Participants were largely satisfied with the level of job responsibility (3.89 ± 0.98), followed by the level of control over job (3.83 ± 1.07), support from co-workers (3.74 ± 0.94), and job security (3.63 ± 1.10). On the other hand, participants were largely not satisfied with salary (2.73 ± 1.15), followed by non-financial incentives (2.80 ± 1.11), and opportunities for career advancement (2.89 ± 1.08).

Raw data (Likert scales) of individual job characteristic items were converted to Rasch scores and overall logit scores were calculated for each item. Negative logit scores indicate no satisfaction and positive logit scores indicate satisfaction. The mean logit scores for overall job satisfaction scores was 0.65 logits (scores ranging from − 1.17 to 1.92 logits; see Table 2). Given that the higher the positive logit score, the higher is the satisfaction, overall job satisfaction was high among optometrists in Ghana.

**Table 2:** Rasch Logit Scores for Determinants of Job Satisfaction among Optometrists in Ghana

Job characteristic Items	N	Minimum	Maximum	Mean	SD
Salary	214	− 0.17	2.92	1.13	0.94
Non-Financial Incentives	214	0.01	3.10	1.36	0.90
Job Security	214	− 1.15	1.94	0.89	0.89
Workplace Equipment	214	− 3.42	− 0.33	− 1.64	0.93
Supervision	214	− 0.60	2.49	1.36	0.90
Encouragement	214	− 0.78	2.31	0.85	0.87
Recognition	214	− 1.43	1.66	0.59	0.86
Responsibility to Work	214	− 2.34	0.75	− 0.08	0.78
Task Variety	214	− 1.70	1.39	0.30	0.76
Workload	214	− 0.26	2.83	1.62	0.82
Control	214	− 2.11	0.98	0.09	0.86
Support from Co-Workers	214	− 2.13	0.96	0.01	0.76
Continuing Education Opportunities	214	− 1.45	1.64	0.12	0.95
Career Advancement Opportunities	214	− 1.57	1.52	− 0.16	0.88
Overall Job Satisfaction	214	− 1.17	1.92	0.65	0.83

N, number of participants; SD, Standard Deviation

### Factors associated with job satisfaction among optometrists in Ghana

Bivariate simple linear regression analyses (see Table 3) showed that the variables significantly associated (*p* < 0.05) with overall job satisfaction were location of workplace, practice setting, number of working hours, Good work-life balance, Salary, Non-Financial Incentives, Job Security, Workplace Equipment, Supervision, Encouragement, Recognition, Responsibility to Work, Task Variety, Workload Control, Support from Co-Workers, Continuing Education Opportunities, and Career Advancement Opportunities.

**Table 3:** Bivariate and multivariate linear regression analyses of factors associated with overall job satisfaction using rasch logit scores

Characteristic	Overall job satisfaction					
	Simple regression			Multiple regression		
	Unstandardized co-ef (β)	S.E	*p* value	Unstandardized co-ef (β)	S.E	*p* value
Age (years)	0.007	0.009	0.449			
Sex: male versus female	0.188	0.126	0.158			
*Marital status*						
Single	Ref					
Married	0.023	0.117	1.000			
Divorced	− 0.061	0.326	1.000			
Number of children	0.088	0.045	0.052			
*Location of work place*						
Urban versus Rural	0.395	0.143	0.006	− 0.058	0.091	0.530
*Practice Setting*				0.058	0.042	0.166
Government	Ref					
CHAG/NGO	− 0.369	0.158	0.204			
Private	− 0.543	0.129	*p* < 0.0005			
Academic	− 0.859	0.259	0.011			
Others	− 0.865	0.467	0.656			
Working hours per week	0.017	0.005	*p* < 0.0005	0.002	0.003	0.422
Work experience (years)	0.005	0.012	0.714			
*Good work- life balance*						
Yes versus No	0.592	0.133	*p* < 0.0005	0.288	0.088	0.001
Salary	0.544	0.047	*p* < 0.0005	0.222	0.049	*p* < 0.0005
Non-Financial Incentives	0.444	0.055	*p* < 0.0005	0.080	0.047	0.091
Job Security	0.496	0.054	*p* < 0.0005	0.025	0.053	0.635
Workplace Equipment	0.361	0.056	*p* < 0.0005	0.002	0.046	0.970
Supervision	0.550	0.051	*p* < 0.0005	0.117	0.058	0.044
Encouragement	0.505	0.056	*p* < 0.0005	0.045	0.054	0.409
Recognition	0.577	0.053	*p* < 0.0005	0.099	0.063	0.118
Responsibility to Work	0.571	0.061	*p* < 0.0005	0.041	0.075	0.583
Task Variety	0.561	0.064	*p* < 0.0005	− 0.057	0.073	0.434
Workload	0.493	0.060	*p* < 0.0005	0.058	0.053	0.270
Control	0.622	0.051	*p* < 0.0005	0.139	0.071	0.052
Support from Co-Workers	0.643	0.060	*p* < 0.0005	0.096	0.068	0.158
Continuing Education Opportunities	0.459	0.050	*p* < 0.0005	0.138	0.057	0.017
Career Advancement Opportunities	0.418	0.057	*p* < 0.0005	− 0.018	0.064	0.777
(constant)				− 0.472	0.251	0.062

F (18, 195) = 24.89; R-squared = 0.697, *p* < 0.0005; S.E., Standard Error of Unstandardized co-efficient (β)

On including the variables that were found to be significant in the bivariate regression models, the multiple regression analyses (see Table 3; F (18, 195) = 24.89; R-squared = 0.697, *p* < 0.0005) showed that Good work-life balance ( Unstandardized co-efficient (β) = 0.288, *p* = 0.001), Salary (β = 0.222, *p* < 0.0005), Supervision (β = 0.117, *p* = 0.044), and Continuing Education Opportunities (β = 0.138, *p* = 0.017) were all significantly associated with higher levels of overall job satisfaction.

## Discussion

This novel study highlights job satisfaction and its associated factors among optometrists in Ghana. The mean score for the overall perception of job satisfaction reported by optometrists in Ghana was 3.36. Majority of optometrists (74.3%) in Ghana reported that they were largely satisfied with their jobs. The main factors associated with overall job satisfaction were good work-life balance, salary, supervision, and Continuing Education Opportunities.

Job satisfaction levels among healthcare professionals have been studied extensively in many different countries, although limited data exist in Africa. In our study, we found job satisfaction level among optometrists in Ghana to be 74.3%. Our finding was found to be almost similar to the level of job satisfaction among optometrists in the United Kingdom (80%) [35]. It is however noteworthy that job satisfaction among healthcare professionals has been studied in the Ghanaian population [5, 36–38], although not specifically among optometrists. A cross-sectional study by Bonenberger et al. [5] similarly reported an overall job satisfaction mean score of 3.15 (out of 5) among healthcare professionals in Ghana. The study had a total of 256 healthcare professionals who were systematically sampled from most public health facilities across the Eastern region of Ghana. However, job satisfaction levels were assessed using the Measure of Job satisfaction (MJS) and the Job Descriptive Index (JDI) tools which were validated by Rouleau et al. [39]. Another cross-sectional study by Boafo [37] also reported an overall job satisfaction mean score of 3.19 (out of 5) among nurses in Ghana. However, a total of 592 qualified nurses were recruited in the study, with job satisfaction levels being assessed by the MJS tool only. The use of different instruments (MJS and JDI) to assess the levels of job satisfaction among other healthcare professionals, as opposed to the use of the validated tool by Paudel et al. [33] which is purposely designed to assess job satisfaction level among eyecare professionals, may have accounted for the slightly higher results obtained among optometrists in our study.

Our study found salary to be significantly associated with overall job satisfaction. This was consistent with a similar survey conducted among the optometric workforce in the United Kingdom [35]. Other studies in Africa have also reported lowest mean score for salary/remuneration in relation to job satisfaction among healthcare professionals [3, 40, 41]. In Ghana, salary/remuneration is perceived to be low among healthcare professionals [17]. Lower financial rewards to these healthcare professionals, including optometrists, may result in low work performance and/or productivity [36], hence the need for stakeholders to adopt policies aimed at enhancing renumeration/salary levels and reducing job turnover among optometrists [42].

The role of supervisory support from organizational leaders has proven to be essential in alleviating health professionals’ job turnover and enhancing overall job satisfaction [43]. Healthcare professionals perceive supervisory support as an important indicator for decreasing work-related emotional exhaustion and improving their work performance [44]. Emotional exhaustion or stress is regarded as a risk factor for health professionals’ wellbeing and health. In Ghana, a study by Yeboah et al. [45] showed that supervisory support given to health professionals, including optometrists, played a pivotal role in their management of work-related stress. Thus, effective clinical supervision of allied healthcare professionals (e.g. optometrists) ultimately ensures continuous professional development and wellbeing, enhance routine clinical tasks, facilitate the delivery of safe and quality eyecare services, and enhance overall job satisfaction [46]. There is therefore the need to develop and implement efficient optometry-specific guidelines and policies on clinical supervision in Ghana, through the leadership of the GOA and AHPC, to support optometrists in their professional role.

The tendency for healthcare professionals to be satisfied at their workplaces may depend on the level of control they have over their work environment. In the United Kingdom, the level of autonomy or independence in job practice was the second most important factor for job satisfaction among optometrists [35]. Lack of control over job, exhibited by some health professionals, signifies a limitation to their sense of discretion and autonomy at their workplaces. Working conditions are generally improved when healthcare professionals possess an appreciable level of command over their work environment. Additionally, it has been shown that lack of job control is a precursor to workload and job burnout or stress [47]. In Australia, work-related stress among optometrists was due to factors such as workload and patient-related clinical problems [14]. However, the mitigating effect of job control over workload and work-related stress has been demonstrated in a study by Portoghese et al. [48]. Thus, a good control over an optometrist’s job in Ghana may offer him/her the opportunity to shape his/her work environment (reducing workload and stress), and subsequently hinder turnover intentions, and promote high levels of job satisfaction [49]. Optometrists in Ghana are therefore likely to remain at their workplaces if issues concerning their level of control over job are addressed.

Social desirability bias was minimized in our study through the following means. First, online questionnaires (via Google forms) were self-administered and allowed for privacy. Therefore, responses from our online mail survey cannot be biased by interviewer presence. Anonymity of respondents was assured by storing responses and identification number in different files (thus, not allowing the linking between responses and identifiers). Secondly, trained field staff, who served as Research Assistants, administered face-to-face interviews to respondents in our study. Furthermore, the study employed a psychometrically validated job satisfaction instrument developed by Paudel et al. [33] for use among eyecare professionals only (such as optometrists). This robust instrument improved the quality of psychometric measures in our study, hence minimizing social desirability bias. Moreover, regarding the questionnaire used in our study, closed-ended questions were used where participants had to indicate their responses on a Likert scale. This reduced the tendency to generate social desirability bias***.***

The strength of this study includes the use of a previously validated questionnaire for data collection among eyecare professionals, which was adapted from Paudel et al. [33]. This instrument provides good precision in measuring the level of job satisfaction among eye care professionals such as optometrists. Secondly, to the best of our knowledge, this survey was the first (novel) study assessing job satisfaction and its associated factors among optometrists in Ghana. However, the cross-sectional nature/design of this study makes it difficult to establish a cause-effect relationship between job satisfaction and its associated factors. This serves as a limitation for this study. Practically, the busy schedule of optometrists in their clinical settings, constraints in follow-up, inadequate resources, unstable/poor internet networks (especially in rural communities), and other official engagements by some optometrists made it impossible to achieve a much higher response rate in our study. In Ghana, combined response rates for surveys among optometrists, using both online and printed questionnaires, are 33.5% [50] and 46% [51]; which highlights the fact that participants’ responses to surveys remains a key challenge, especially among participants from a single profession (i.e. optometrists). Future studies must be conducted to ascertain the association between communication, social relations at the workplace, and work safety, and job satisfaction among optometrists in Ghana.

## Conclusions

On a 1–5-point scale (1—very dissatisfied; 5—very satisfied), the mean score of the overall perception of job satisfaction among optometrists was 3.36 (*satisfied*). Overall, 74.3% of optometrist reported that they were satisfied with their jobs. Good work-life balance, salary, supervision, and Continuing Education Opportunities were significantly associated with overall job satisfaction. Findings from this study are very essential to employers, policymakers, healthcare managers, and other stakeholders of the eyecare sector in understanding the impact of job satisfaction on optometrists’ retention and productivity at workplaces, as well as policy revision on the national planning and management of human resources for eye health (particularly optometrists) in Ghana and across Africa.

## Data Availability

The dataset used and/or analysed during the current study are available from the corresponding author on reasonable request.

## Abbreviations

WHO: World Health Organization
SDG: Sustainable Development Goals
GOA: Ghana Optometric Association
AHPC: Allied Health Professions Council of Ghana
KNUST: Kwame Nkrumah University of Science and Technology
OR: Odds ratio
CI: Confidence interval
SD: Standard deviation
OD: Doctor of Optometry
MJS: Measure of Job satisfaction
JDI: Job Descriptive Index
